# Postauricular Skin Mycobiome Profiles in Atopic Dermatitis Treated With Dupilumab or Cyclosporine A: A Descriptive Case Series

**DOI:** 10.1111/1346-8138.70083

**Published:** 2025-11-28

**Authors:** Yuta Koike, Hitomi Morisaki, Daisuke Motooka, Mai Matsumoto, Motoi Takenaka, Hiroyuki Murota

**Affiliations:** ^1^ Department of Dermatology Nagasaki University Graduate School of Biomedical Sciences Nagasaki Japan; ^2^ Leading Medical Research Core Unit, Life Science Innovation Nagasaki University Graduate School of Biomedical Sciences Nagasaki Japan; ^3^ Department of Infection Metagenomics, Genome Information Research Center, Research Institute for Microbial Disease Osaka University Suita Japan

**Keywords:** atopic dermatitis, cyclosporine, dupilumab, skin mycobiome

## Abstract

Atopic dermatitis (AD) essentially exhibits dysbiosis of skin fungal microbiome, mycobiome, characterized by depletion of *Malassezia*. The effects of recent systemic therapies for AD on skin mycobiome were not understood enough. We examined changes of skin mycobiome before and after systemic treatments with anti‐IL‐4Rα antibody (dupilumab: DUP) and calcineurin inhibitor (cyclosporine, CyA). Swab samples from postauricular areas in 19 AD patients treated with dupilumab (*n* = 13) and cyclosporine (*n* = 6) were collected before and 4–8 weeks after starting each treatment. Fungal DNA was amplified from the samples and sequenced with ITS1 metagenomic analysis, and taxonomic classification was performed. Fungi belonging to total 89 genera were detected. The share of the fungus was most occupied by *Malassezia* (81.3%), followed by *Aspergillus* (3.7%), and *Trametes* (1.1%) before DUP and CyA treatment, and occupied by *Malassezia* (87.3%), followed by *Aspergillus* (1.9%), and *Candida* (1.7%) after treatment. Three AD patients whose ratio of *Malassezia* in the skin mycobiome was under 50%, showed an exploratory increase of *Malassezia* after treatments (before 17.3%, after 67%). Analysis of the *Malassezia* species revealed an increase in *M. restricta* (before 70.5%, after 79.5%) and a decrease in *M. globosa* (before 23.9%, after 16.1%). No consistent patterns distinguishing DUP and CyA were observed. Systemic treatment with DUP and CyA was associated with shifts toward higher *Malassezia* abundance and modulation between *M. restricta* and *M. globosa.* These findings are exploratory and require validation in larger controlled studies.

## 1 Introduction

The skin is inhabited by a diverse community of microorganisms, including bacteria, fungi, and viruses, which maintain human health by producing antibacterial peptides, forming biofilms, and inhibiting pathogen invasion [1]. Additionally, the skin microbiome comprises a complex ecosystem in which the physiochemical conditions produced by the resident microbiome interact with host biology [2]. When the intact skin microbiome breaks down, the abnormal microbiome condition is called dysbiosis, sometimes leading to the exacerbation of host skin diseases [3].

Atopic dermatitis (AD) is a common, chronic inflammatory skin disease, in which patients' quality of life are severely disturbed by inpatient itching [4]. The etiology of AD is multifactorial and has not yet been fully elucidated. One of the contributing factors that establish AD skin is dysbiosis of the skin bacterial microbiome characterized by a reduction in microbial diversity, overgrowth of *Staphylococcus aureus* , and a relative reduction of commensal species [5]. In recent years, the skin fungal microbiome, known as the name of skin mycobiome, has also been highlighted in skin diseases. The human skin mycobiome shows relative monotony, in which the genus *Malassezia* dominates the skin mycobiome, with only minor contributions from other fungi under steady‐state conditions [6]. The AD skin mycobiome is characterized by depleted amounts of *Malassezia* and a higher abundance of filamentous fungi [7].

In recent years, biological drugs targeting specific inflammatory cytokines have been used to improve inflammatory skin disorders, such as interleukin (IL)‐4, IL‐13, and IL‐31 for AD and tumor necrosis factor (TNF) α, IL‐17, and IL‐23 for psoriasis. These treatments not only attenuate skin inflammation activity but also influence the bacterial microbiome in humans. For example, AD treatment with an IL‐4RA inhibitor namely dupilumab (DUP) shifts the bacterial microbiome toward healthy skin flora [8]. Anti‐IL‐23p40 antibody therapy for psoriasis increased the β‐diversity of the skin bacterial microbiome to after treatment [9]. A patient with psoriasis who had been treated with an anti‐IL‐17A antibody developed AD‐like eczema in exchange for complete remission of psoriasis, which might be associated with skin bacterial dysbiosis [10]. Focusing on the skin mycobiome, a few research are carried out in psoriasis. Treatment with anti‐IL‐23p19 antibodies for psoriasis decreased the genus *Malassezia* on the antecubital fossa, whereas treatment with TNF‐α, IL‐17, and IL‐23p19 inhibitors did not affect the mycobiome on the postauricular skin [11, 12]. The features of skin mycobiome in Asian AD skin, which is characterized by not only Th2 but also Th17 signal involvement [13], and the effects of biological therapies for AD skin mycobiome were not sufficiently investigated.

Here, we conducted a descriptive analysis of the effects of systemic treatment with DUP and cyclosporine A (CyA) on the AD skin mycobiome. Swab samples were collected from postauricular skin before and after treatment DUP and CyA. Postauricular skin, a seborrheic and fungal‐rich skin, was selected as the sample collection site because this study aimed to elucidate the direct influence of systemic therapy on steady‐state AD skin. The effects of these treatments on skin mycobiomes were examined using ITS1 metagenomic sequencing. Finally, analysis of taxonomic diversity provided exploratory insights into the impact of systemic AD treatments on the skin mycobiome.

## 2 Methods

### 2.1 Study Subjects

Nineteen patients with AD in whom 13 treated with DUP, and six treated with CyA at the Department of Dermatology and Allergology, Nagasaki University Hospital, were enrolled. The participants were provided oral informed consent or the opportunity to opt out under protocols approved by the ethics committee of the Nagasaki University Hospital (22071112). The information of sex, current age, age at AD onset, past history, comorbidities, history of systemic treatment, blood test before treatment (counts for white blood cells and eosinophils, serum, IgE, and TARC), and the Eczema Area and Severity Index (EASI) score are retrospectively collected and are summarized in Table 1. The EASI score in a CyA‐treated patient and IgE and TARC in two DUP‐treated patients had missing data and were excluded from the Table.

**TABLE 1:** Patient characteristics.

Patient numbers	Male/Female	Age^a^	EASI^a^	IgE^a^ (IU/ml)	TARC^a^ (pg/ml)
DUP (*n* = 13)	10/3	41.8 ± 12.9 (18–68)	27.2 ± 9.5 (16.4–45)	10 769 ± 8435 (266–23 952)	9039 ± 9291 (236–28 010)
CyA (*n* = 6)	6/0	35.7 ± 11.9 (22–53)	27.1 ± 13.2 (17.7–36.6)	15 841 ± 13 586 (2316–37 602)	5091 ± 2543 (2386–8343)
ALL (*n* = 19)	16/3	39.8 ± 12.6 (18–68)	27.1 ± 9.5 (16–45)	12 354 ± 10 126 (266–37 602)	7805 ± 7927 (236–28 010)

Abbreviations: CyA, Cyclosporine A; DUP, Dupilumab; EASI, Eczema Area and Severity Index; TARC, Thymus and activation‐regulated chemokine.

^a^ average ± standard deviation (range).

### 2.2 Sample Collection

Dry swab samples were collected from each patient by rubbing the postauricular area over 50 times using culture swabs (BD BBD culture swab plus; BD, Japan) before treatment with DUP or CyA and after 4–8 weeks of treatment. There were no standardized conditions before sampling. The swab samples were stored at −80°C until DNA extraction.

### 2.3 Fungal ITS1 Deep Sequencing, Bioinformatic Analysis, and Taxonomic Assignment

Fungal ITS1 deep sequencing, bioinformatic analysis, and taxonomic assignment were performed as described previously [11]. Briefly, DNA was extracted from swab samples using the KURABO PI‐1200 DNA isolation system (Kurabo, Japan). Library preparation involved a two‐step PCR method with the primer set ITS1‐F (5′‐CTTGGTCATTTAGAGGAAGTAA‐3′) and ITS2 (5′‐GCTGCGTTCTTCATCGATGC‐3′) targeting the fungal ITS1 region, along with the Nextera XT Index Kit v2 (Illumina). Paired‐end sequencing of 301 bp amplicons was then carried out on a MiSeq instrument (Illumina). The resulting paired‐end reads were merged, filtered, and denoized using DADA2. Taxonomic assignment was conducted using the QIIME2 feature‐classifier plugin applied to the ntF‐ITS1 database, and all raw sequencing data were processed within the QIIME2 pipeline (version 2020.2) bioinformatics environment [14].

### 2.4 Statistical Analysis

Given the exploratory nature of this study, no formal hypothesis testing, sample size calculation or power estimation was performed. Data were summarized using descriptive statistics and visualized to illustrate variability without implying excessive precision. The α diversity (Shannon's diversity index) was calculated using the bioinformatics pipeline QIIME2 (version 2020.2). Results were expressed as mean ± standard error (SE). Data processing and visualization were performed using Microsoft Excel and GraphPad Prism 8 (GraphPad, San Diego, CA, USA).

## 3 Results

### 3.1 Skin Mycobiome of Individual Samples Before and After Treatment With DUP or CyA

**FIGURE 1:**
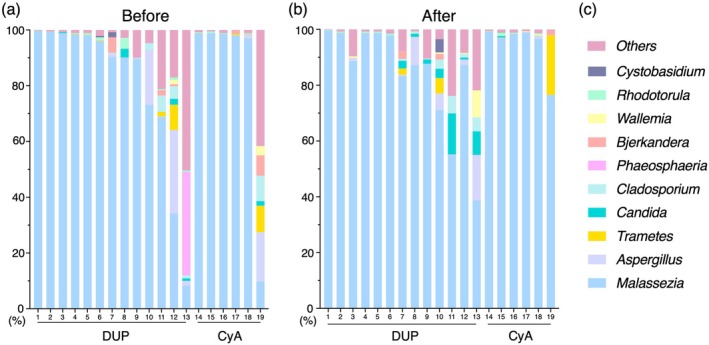
Analysis of the skin mycobiome of atopic dermatitis patients was performed using swab samples collected from postauricular skin at two time points; (a) before and (b) after treatment with DUP and CyA. Numbers under the horizontal line are individual patients, 1–13 are treated with DUP and 14–19 are treated with CyA, corresponding before and after treatments. (c) Vertical bars represent the ratio of individual fungi in the mycobiome with fungal names matched to their color. CyA, Cyclosporine A; DUP, Dupilumab.

The mycobiome on the postauricular skin of AD patients collected before and 4–8 weeks after treatment with DUP or CyA was analyzed. From the sequence results of all swab samples, a total of 89 genera of fungi were detected, of which the genus *Malassezia* had the highest average individual fungal composition (84.3%, Table S1). The details of fungal genera populations per sample derived from before treatment are shown in Figure 1a, and those after treatment with DUP or CyA are shown in Figure 1b, with the color bars corresponding to the top 10 genera detected before treatment (Figure 1c). Genus *Malassezia* is occupied over 50% on mycobiome in 16/19 samples before treatment and 18/19 samples after treatment.

### 3.2 Genus Level Analysis of Skin Mycobiome Between Before and After Treatment

**FIGURE 2:**
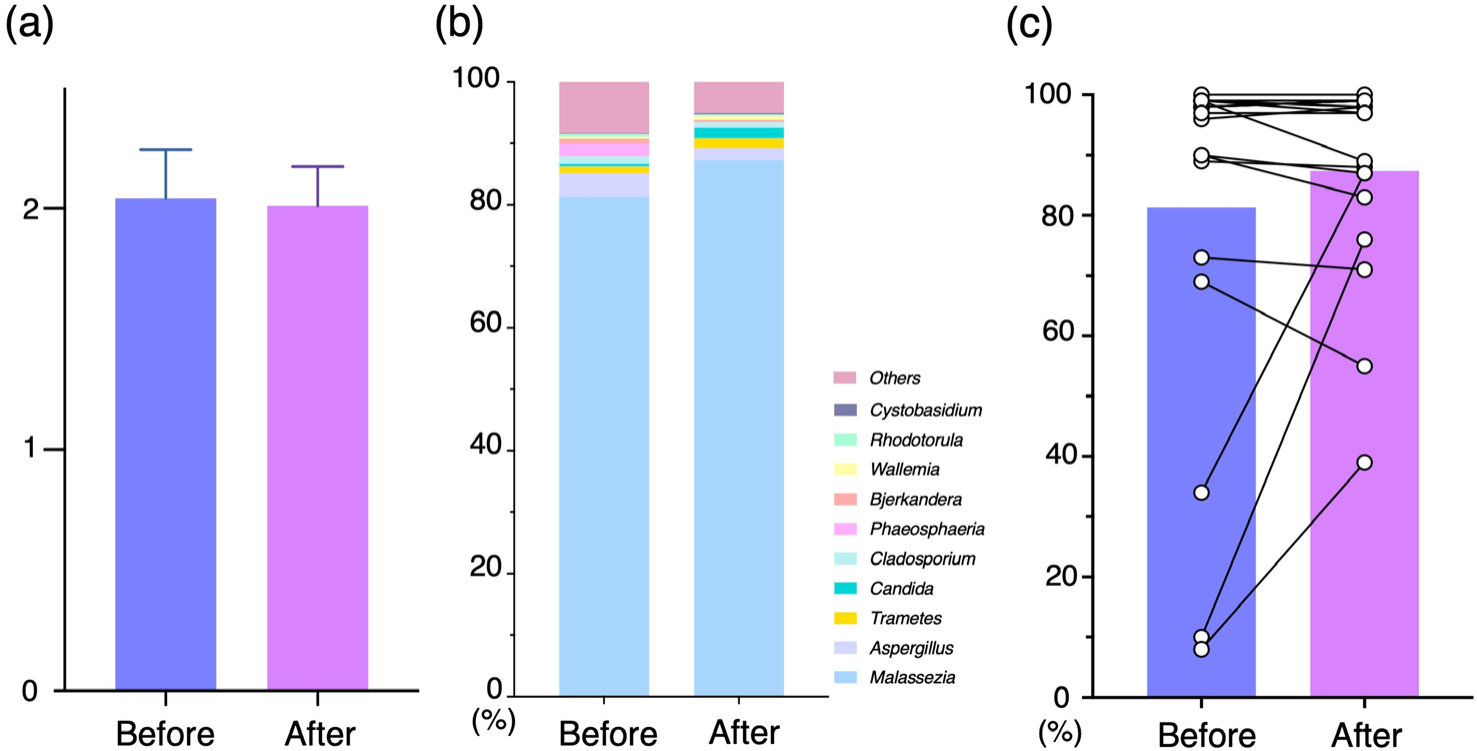
(a) Averages of an α‐diversity index, the Shannon index, of the skin mycobiome in AD showed similar results before and after systemic treatments. (b) The average percentage of individual fungi showed a mild increase in *Malassezia*. (c) Composition of *Malassezia* in the skin mycobiome before and after systemic treatment is shown with bars (average) and paired dots (individual AD patients). AD, Atopic dermatitis.

We integrated and comprehensively analyzed individual skin mycobiome data to identify the features after systemic AD treatment. An index of α diversity, the Shannon index, was similar before (2.04 ± 0.87) and after treatment (2.01 ± 0.70, Figure 2a). A β diversity analysis using PCoA with weighted UniFrac distance and PCA analysis, showed no consistent separation between the groups (Figure S1). A comparison of the mean ratios of individual fungi constituting the skin mycobiome both before and after treatment is shown in Figure 2b. The average percentages of the genera in the before‐treatment samples were as follows: *Malassezia* (81.3%), *Aspergillus* (3.7%), *Phaeosphaeria* (1.9%), *Trametes* (1.1%), and *Candida* (0.4%), and those in after‐treatment samples were as follows: *Malassezia* (87.3%), *Aspergillus* (1.9%), *Candida* (1.7%), *Trametes* (1.5%), and *Cladosporium* (0.9%). There were no specific genera whose average rates were clearly changed before and after treatment, whereas the genus *Malassezia* showed mild increase the relative abandance from before to after treatment (81.3% ± 6.9% and 87.3%± 3.8%, respectively). Three patients whose share of genus *Malassezia* in skin mycobiome was under 50% before treatment, showed an increase in the relative abandance of *Malassezia* after systemic treatment (average: before 17.3% ± 14.4%, after 67.3% ± 25.1%, Figure 2c). When separating the mycobiome results into DUP or CyA treatment groups, the average rate of genus *Malassezia* similarly increased after treatment in both groups (DUP, from 80.3% to 84.0%; CyA, from 83.4% to 94.3%; Figure S2). Thus, systemic treatment for AD was associated with a tendency toward increased the genus *Malassezia* in the skin mycobiome, especially in patients with a low composition of the genus *Malassezia* on their skin, which suggests a possible movement toward *Malassezia*‐dominant state.

### 3.3 *Malassezia* Species in Individual Samples Before and After Treatment With DUP and CyA

**FIGURE 3:**
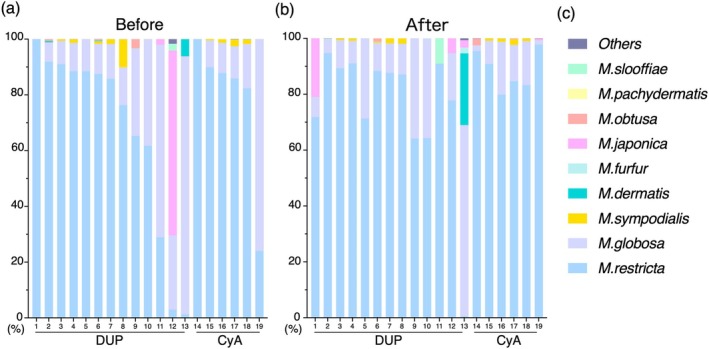
Analysis of *Malassezia* species in the postauricular area of atopic dermatitis patients is performed with samples taken (a) before and (b) after treatment with DUP and CyA. Numbers under the horizontal line are individual patients; 1–13 are treated with DUP and 14–19 are treated with CyA, corresponding before and after treatments. (c) Vertical bars represent the ratio of individual *Malassezia* species in the whole of *Malassezia* with species names matched to their color. CyA, Cyclosporine A; DUP, Dupilumab.

Next, we focused on the details of each *Malassezia* species in the genus *Malassezia*. The individual populations before and after treatment are shown in Figure 3a and Figure 3b, respectively, with the names of detected nine species of *Malassezia* (Figure 3c). Among samples before treatment, *M. restricta* is the most dominant in 15/19 samples (78.9%), followed by *M. globosa* in 3/19 samples, and *M. japonica* in 1/19 sample. Likewise, among the samples after systemic treatment, *M. restricta* was the most dominant in 18/19 samples (94.7%), and *M. globosa* was the most in only one sample. These results suggest that *Malassezia* species on the postauricular skin in AD patients are dominated by *M. restricta*, in which several samples especially before treatment disrupts the domination.

### 3.4 Treatments With DUP and CyA Modulate the Composition Balance Between *M. Restricta* and *M. globosa*

**FIGURE 4:**
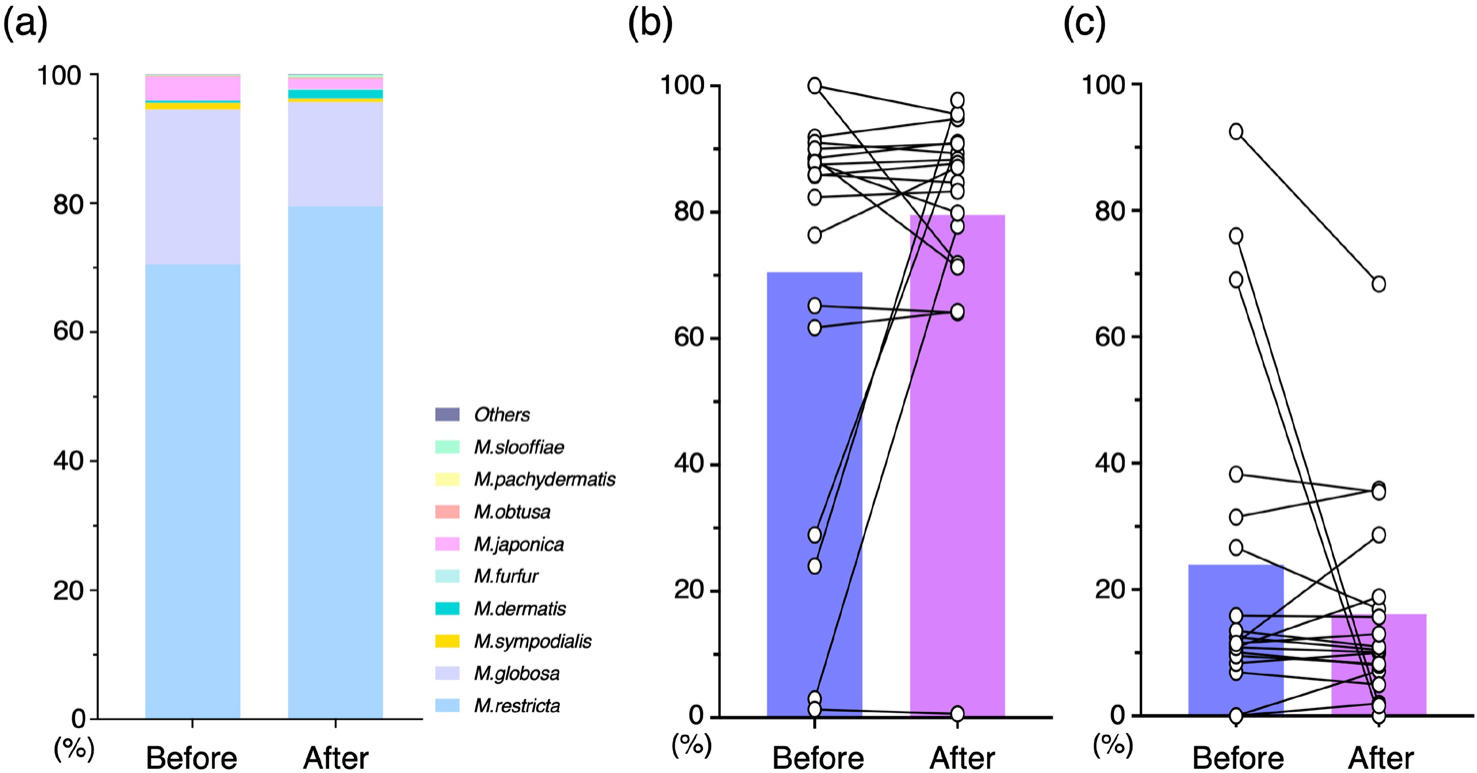
(a) Average percentage of individual *Malassezia* species in the total *Malassezia* showed a mild increase in *M. restricta* and a mild decrease of *M. globosa* . Composition of (b) *M. restricta* and (c) *M. globosa* in the total *Malassezia* before and after systemic treatment is shown with bars (average) and paired dots (individual atopic dermatitis patients).

The average ratios of the *Malassezia* species before and after treatment were shown in Figure 4a. *M. restricta* was the most dominant in samples before treatment (70.5%), followed by *M. globosa* (23.9%), *M. japonica* (3.5%), *M. sympodialis* (1.0%), and *M. dermatis* (0.3%). Similarly, *M. restricta* showed the most dominant after treatment (79.5%), followed by *M. globosa* (16.1%), *M. japonica* (1.5%), *M. dermatis* (1.3%), and *M. sympodialis* (0.5%). Focusing on the individual ratio of fungi, *M. restricta* increased its ratio (from 70.5% ± 31.8% to 79.5% ± 21.5%) and *M. globosa* decreased its ratio (from 23.9% ± 26.6% to 16.1% ± 16.3%) after treatment. When separating the treatment groups into DUP and CyA treatments, we obtained similar results on each treatment (Figure S3). Four patients whose ratio of *M. restricta* in total *Malassezia* species was under 50% tended to increase that of *M. restricta* after treatment (before 14.2% **±** 7.1%, after 66.7% **±** 22.4%, Figure 4b). Similarly, three patients whose ratio of *M. globosa* in *Malassezia* species was over 50% in the mycobiome tended to have decreased *M. globosa* after treatment (before 79.1% **±** 6.9%, after 23.2% **±** 22.5%, Figure 4c). Thus, DUP and CyA treatment for AD may be associated with modulation of the balance of *Malassezia* species characterized by an increase in *M. restricta* and a decrease in *M. globosa* , especially in patients with disrupted *M. restricta* domination to *M. globosa* on their skin.

## 4 Discussion

In this study, we evaluated the skin mycobiome of AD patients systemically treated with DUP and CyA. Paired skin swab samples before treatment and 4–8 weeks after starting treatment were obtained and analyzed to describe the prevalence of different genera within the mycobiome. The results indicated that systemic treatments for AD were associated with tendency toward increased *Malassezia* in the skin mycobiome, especially in cases where the ratio of *Malassezia* was less than 50% before treatment. Focusing on *Malassezia* species, the ratio of *M. restricta* tended to increase after systemic treatment, in contrast to that of *M. globosa* which tended to decrease. No consistent patterns distinguishing DUP and CyA in the skin mycobiome were observed. Thus, systemic treatments for AD may be associated with the skin mycobiome characterized by a tendency toward increased *Malassezia*, especially *M. restricta*, indicating possible modulation of the skin mycobiome in AD skin.

Previous studies have suggested fungal dysbiosis in the skin of AD patients. The most characteristic fungal community of AD skin is the decrease in the genus *Malassezia* [7]. Since the genus *Malassezia* is essentially a dominant fungus for healthy skin, its depletion is an irregular condition for the skin that may allow the proliferation of pathogenic microorganisms and then prompt skin inflammation. Our results suggest that AD patients, whose genus *Malassezia* on the skin was depleted before treatment, can expand the genus *Malassezia* on their skin after treatment with DUP and CyA, which may be a modulation from the fungal dysbiosis of the AD skin. Genus *Candida*, that is usually live in the mucosal area on human, are found in several samples both before and after treatment. *Candida* is more frequently identified on AD skin than on healthy skin [15]. Our previous investigation of the skin mycobiome, in which we collected postauricular swab samples from 19 psoriasis patients using the same method of the current research, revealed that the genus *Candida* DNA was not detected at all even after treatment with anti‐TNFα antibody or anti‐IL‐17A antibody [12]. The presence of *Candida* in the postauricular skin might be a feature of AD skin that is not acquired even with immunosuppression by TNF or IL‐17.

The major component of *Malassezia* species on human skin is *M. restricta*, followed by *M. globosa* . These *Malassezia* species are commensal and usually not harmful to human skin, although overgrowth of these species sometimes causes skin diseases, such as seborrheic dermatitis and pityriasis versicolor [16]. The ratio of the predominant *Malassezia* species associates severity of AD lesions. Ratio of *M. restricta* is low and that of *M. globosa* is high in severe AD skin in comparison with healthy skin and mild AD skin [17, 18]. The inflammation mechanisms of Malassezia is thought to have several ways *Malassezia* can penetrate the defects in the skin barrier subsequently be recognized by pattern recognition receptors and initiate skin inflammation [19, 20]. In addition, the genus *Malassezia* has been suggested to provoke an allergic reaction and exacerbate the inflammatory response in AD [21]. Especially, *M. globosa* produces a protein namely MGL_1304 and dissolve it into human sweat. Recombinant MGL_1304 induces histamine release from basophils from patients with AD [22], which can explain the reverse phenomenon of the ratio of *M. restricta* and *M. globosa* in severe AD skin. Our results showed trends in which some swab samples from the AD skin showed a low ratio of *M. restricta* and a high ratio of *M. globosa* , and the ratios appeared to move toward a normal balance after systemic treatment for AD, suggesting possible modulation of *Malassezia* composition. These exploratory observations may imply a potential reduction of type 1 allergic responses related to proteins produced by *M. globosa* .

The variety of *Malassezia* species is also characteristic on AD. The relative abundances of *M. dermatis* and *M. sympodialis* were increased in AD lesions, and some species of *Malassezia* such as *Malassezia slooffiae*, *Malassezia obtusa* and *Malassezia yamatoensis*, were detected exclusively in the AD samples [23, 24]. Our results showed nine *Malassezia* species on the postauricular skin where is seborrheic and is relatively steady skin, while only four species were detected in psoriasis patients [12]. The multifarious inhabitant *Malassezia* species might be a feature of dysbiosis in the AD skin. However, our results did not show clear changes in the diversity of *Malassezia* species.

We observed no consistent differences in the skin mycobiome between the DUP and CyA treatments. A recent report on the skin bacterial microbiome revealed that DUP, but not CyA treatment, improved dysbiosis in patients with moderate‐to‐severe AD, in which *S. aureus* was reduced toward healthy control levels and α diversity was increased only in the DUP treatment group [8]. One of the reasons why DUP could improve the skin bacterial microbiome might be that DUP is an immunomodulator that ameliorates type 2 allergies in contrast to CyA as a broad immunosuppressant. The response of the skin microbiome to systemic treatment of AD might depend on the type of microorganisms, bacteria or fungi.

Our study provided the descriptive analysis regarding alterations in the skin mycobiome in patients with AD due on systemic therapies. However, this study had several limitations. This study did not include a healthy control group and AD group without systemic treatments. Generally, the skin mycobiome generally remains relatively stable in natural variations [25]. The relatively small cohort size may have limited the ability to detect subtle changes in the mycobiome such as the differences between DUP and CyA treatments. The sample collection timing after systemic treatments may affect the results of skin microbiome. A study of the skin bacterial microbiome revealed that systemic IL‐23 inhibition therapy for psoriasis initiated a gradual increase in the β‐diversity of the bacterial microbiome from before treatment to after 4 and 28 weeks of treatment [9]. In our current method, samples were collected 4–8 weeks after treatment which might be insufficient to alter the skin mycobiome. Regarding sample collection, washing of the sampling site might have affected the number of microorganisms [26]. Similarily sex and age distribution, disease severity, topical treatments, personal hygiene practices, and cleansing/face‐washing routines should have been standardized or adjusted. These factors can materially influence mycobiome profiles. Despite these limitations, our study provides descriptive insights into alterations of the mycobiome of AD skin after systemic treatment, serving as hypothesis generating evidence that warrants validation in larger, controlled cohorts.

## Funding

This work was supported by the Leading Medical Research Core Unit, Life Science Innovation, Nagasaki University Graduate School of Biomedical Sciences. Japan Society for the Promotion of Science (25K11567). Japan Agency for Medical Research and Development (JP256f0137009).

## Ethics Statement

Protocol was approved by the ethics committee of the Nagasaki University Hospital (22071112).

## Conflicts of Interest

Yuta Koike, Motoi Takenaka, and Hiroyuki Murota received honoraria as a speaker or chair of lectures from Sanofi and Novartis.

## Supporting information


**Figure S1:** β diversity analysis using PCoA with weighted UniFrac distance and PCA analysis.

**Figure S2:** Average proportion of fungal genera in DUP or CyA treatment groups.

**Figure S3:** Average proportion of *Malassezia* species in DUP or CyA treatment groups.

**Table S1:** List of detected fungal genera from skin swab samples.

## Data Availability

The data that support the findings of this study are available from the corresponding author upon reasonable request.

## References

[1] Y. Belkaid and J. A. Segre , “Dialogue Between Skin Microbiota and Immunity,” Science 346, no. 6212 (2014): 954–959, 10.1126/science.1260144. 25414304

[2] E. A. Grice and J. A. Segre , “The Skin Microbiome,” Nature Reviews. Microbiology 9, no. 4 (2011): 244–253, 10.1038/nrmicro2537. 21407241 PMC3535073

[3] L. F. Koh , R. Y. Ong , and J. E. Common , “Skin Microbiome of Atopic Dermatitis,” Allergology International 71, no. 1 (2022): 31–39, 10.1016/j.alit.2021.11.001. 34838450

[4] H. Murota , Y. Koike , H. Morisaki , M. Matsumoto , and M. Takenaka , “Exacerbating Factors and Disease Burden in Patients With Atopic Dermatitis,” Allergology International 71, no. 1 (2022): 25–30, 10.1016/j.alit.2021.10.002. 34764038

[5] A. S. Paller , H. H. Kong , P. Seed , et al., “The Microbiome in Patients With Atopic Dermatitis,” Journal of Allergy and Clinical Immunology 143, no. 1 (2019): 26–35, 10.1016/j.jaci.2018.11.015. 30476499 PMC7163929

[6] K. Findley , J. Oh , J. Yang , et al., “Topographic Diversity of Fungal and Bacterial Communities in Human Skin,” Nature 498, no. 7454 (2013): 367–370, 10.1038/nature12171. 23698366 PMC3711185

[7] R. Tao , R. Li , and R. Wang , “Dysbiosis of Skin Mycobiome in Atopic Dermatitis,” Mycoses 65, no. 3 (2022): 285–293, 10.1111/myc.13402. 34817898

[8] J. Hartmann , L. Moitinho‐Silva , N. Sander , et al., “Dupilumab but Not Cyclosporine Treatment Shifts the Microbiome Toward a Healthy Skin Flora in Patients With Moderate‐To‐Severe Atopic Dermatitis,” Allergy 78, no. 8 (2023): 2290–2300, 10.1111/all.15742. 37032440

[9] M. A. Loesche , K. Farahi , K. Capone , et al., “Longitudinal Study of the Psoriasis‐Associated Skin Microbiome During Therapy With Ustekinumab in a Randomized Phase 3b Clinical Trial,” Journal of Investigative Dermatology 138, no. 9 (2018): 1973–1981, 10.1016/j.jid.2018.03.1501. 29559344

[10] N. Hattori , Y. Koike , and H. Murota , “Skin Microbiome Analysis in a Case of Atopic Dermatitis Induced by an Interleukin 17 Inhibitor Used to Treat Psoriasis,” Journal of Dermatology 50, no. 1 (2023): 104–106, 10.1111/1346-8138.16586. 36117474

[11] Y. Koike , S. Kuwatsuka , D. Motooka , and H. Murota , “Dysbiosis of the Human Skin Mycobiome in Patients Receiving Systemic il‐23 Inhibitors,” Allergology International 74, no. 1 (2025): 72–77, 10.1016/j.alit.2024.06.003. 39307589

[12] Y. Koike , S. Kuwatsuka , K. Nishimoto , D. Motooka , and H. Murota , “Skin Mycobiome of Psoriasis Patients Is Retained During Treatment With Tnf and il‐17 Inhibitors,” International Journal of Molecular Sciences 21, no. 11 (2020): 3892, 10.3390/ijms21113892. 32486022 PMC7312082

[13] S. Noda , M. Suarez‐Farinas , B. Ungar , et al., “The Asian Atopic Dermatitis Phenotype Combines Features of Atopic Dermatitis and Psoriasis With Increased Th17 Polarization,” Journal of Allergy and Clinical Immunology 136, no. 5 (2015): 1254–1264, 10.1016/j.jaci.2015.08.015. 26428954

[14] E. Bolyen , J. R. Rideout , M. R. Dillon , and N. A. Bokulich , “Reproducible, Interactive, Scalable and Extensible Microbiome Data Science Using Qiime 2,” Nature Biotechnology 37, no. 8 (2019): 852–857, 10.1038/s41587-019-0209-9. PMC7015180 31341288

[15] S. M. Edslev , P. S. Andersen , T. Agner , et al., “Identification of Cutaneous Fungi and Mites in Adult Atopic Dermatitis: Analysis by Targeted 18s Rrna Amplicon Sequencing,” BMC Microbiology 21, no. 1 (2021): 72, 10.1186/s12866-021-02139-9. 33663381 PMC7934438

[16] T. Sugita , T. Boekhout , A. Velegraki , J. Guillot , S. Hađina , and F. J. Cabañes , “Epidemiology of Malassezia‐Related Skin Diseases,” in Malassezia and the Skin: Science and Clinical Practice (Springer Berlin Heidelberg, 2010), 10.1007/978-3-642-03616-3_3.

[17] M. Kaga , T. Sugita , A. Nishikawa , Y. Wada , M. Hiruma , and S. Ikeda , “Molecular Analysis of the Cutaneous Malassezia Microbiota From the Skin of Patients With Atopic Dermatitis of Different Severities,” Mycoses 54, no. 4 (2011): e24–e28, 10.1111/j.1439-0507.2009.01821.x. 20002882

[18] E. Zhang , T. Tanaka , M. Tajima , R. Tsuboi , A. Nishikawa , and T. Sugita , “Characterization of the Skin Fungal Microbiota in Patients With Atopic Dermatitis and in Healthy Subjects,” Microbiology and Immunology 55, no. 9 (2011): 625–632, 10.1111/j.1348-0421.2011.00364.x. 21699559

[19] J. Faergemann , “Atopic Dermatitis and Fungi,” Clinical Microbiology Reviews 15, no. 4 (2002): 545–563, 10.1128/CMR.15.4.545-563.2002. 12364369 PMC126862

[20] P. Brodska , P. Panzner , K. Pizinger , and P. Schmid‐Grendelmeier , “Ige‐Mediated Sensitization to Malassezia in Atopic Dermatitis: More Common in Male Patients and in Head and Neck Type,” Dermatitis 25, no. 3 (2014): 120–126, 10.1097/DER.0000000000000040. 24819285

[21] M. Glatz , P. Bosshard , and P. Schmid‐Grendelmeier , “The Role of Fungi in Atopic Dermatitis,” Immunology and Allergy Clinics of North America 37, no. 1 (2017): 63–74, 10.1016/j.iac.2016.08.012. 27886911

[22] T. Hiragun , K. Ishii , M. Hiragun , et al., “Fungal Protein Mgl_1304 in Sweat Is an Allergen for Atopic Dermatitis Patients,” Journal of Allergy and Clinical Immunology 132, no. 3 (2013): 608–615, 10.1016/j.jaci.2013.03.047. 23726042

[23] K. R. Chng , A. S. Tay , C. Li , et al., “Whole Metagenome Profiling Reveals Skin Microbiome‐Dependent Susceptibility to Atopic Dermatitis Flare,” Nature Microbiology 1, no. 9 (2016): 16106, 10.1038/nmicrobiol.2016.106. 27562258

[24] S. H. Han , H. I. Cheon , M. S. Hur , et al., “Analysis of the Skin Mycobiome in Adult Patients With Atopic Dermatitis,” Experimental Dermatology 27, no. 4 (2018): 366–373, 10.1111/exd.13500. 29356103

[25] J. Oh , A. L. Byrd , M. Park , Nisc Comparative Sequencing Program , H. H. Kong , and J. A. Segre , “Temporal Stability of the Human Skin Microbiome,” Cell 165, no. 4 (2016): 854–866, 10.1016/j.cell.2016.04.008. 27153496 PMC4860256

[26] H. H. Kong , B. Andersson , T. Clavel , et al., “Performing Skin Microbiome Research: A Method to the Madness,” Journal of Investigative Dermatology 137, no. 3 (2017): 561–568, 10.1016/j.jid.2016.10.033. 28063650 PMC5468751

