# Supplementary figures and images for: Postauricular Skin Mycobiome Profiles in Atopic Dermatitis Treated With Dupilumab or Cyclosporine A: A Descriptive Case Series
Source: J Dermatol. 2025 Nov 28;53(3):430–6. doi: 10.1111/1346-8138.70083 (PMC12967678; doi:10.1111/1346-8138.70083)

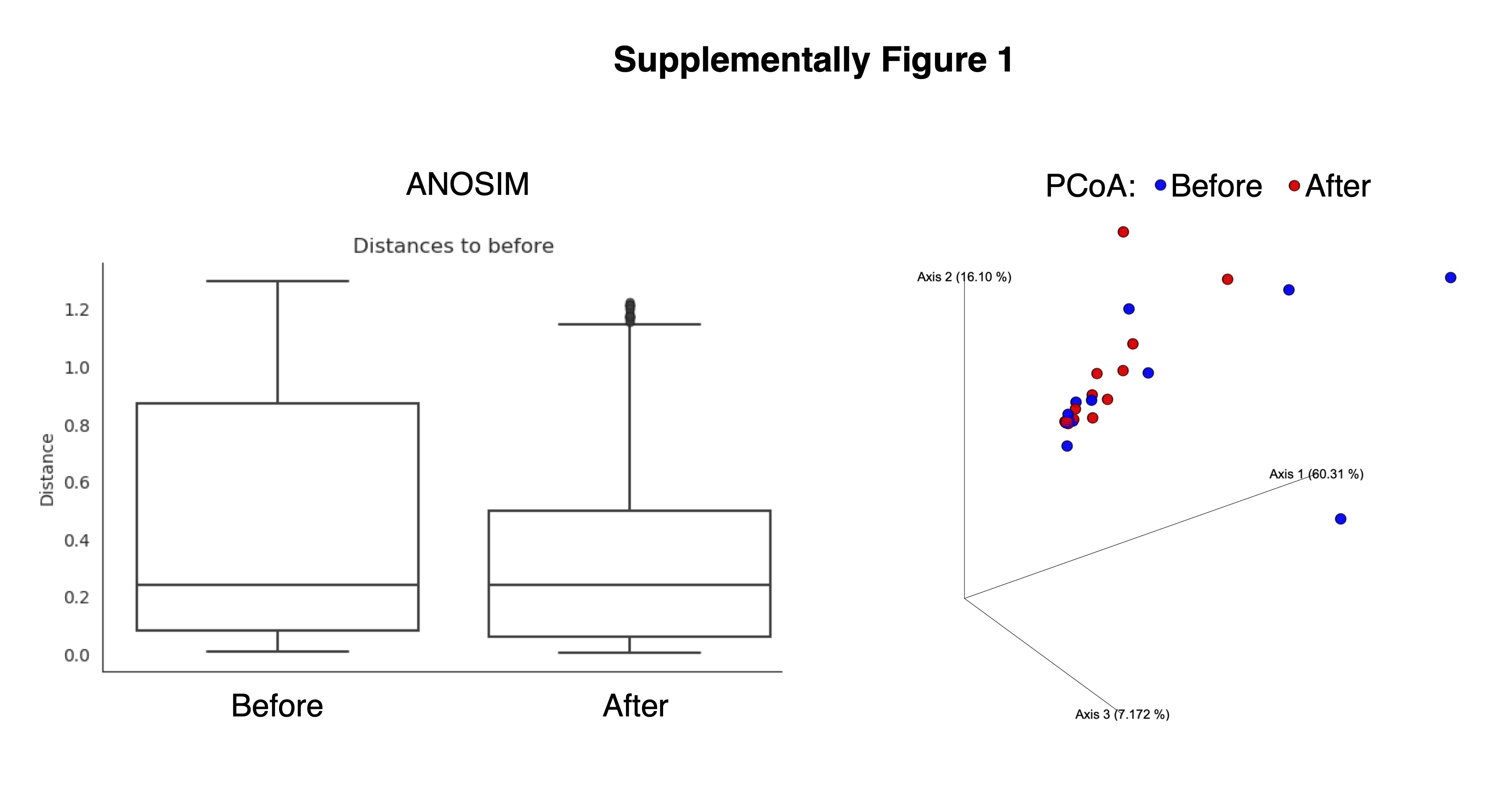

Supplement: Supplementary file 1 — Figure S1: β diversity analysis using PCoA with weighted UniFrac distance and PCA analysis. [file JDE-53-430-s001.jpg]

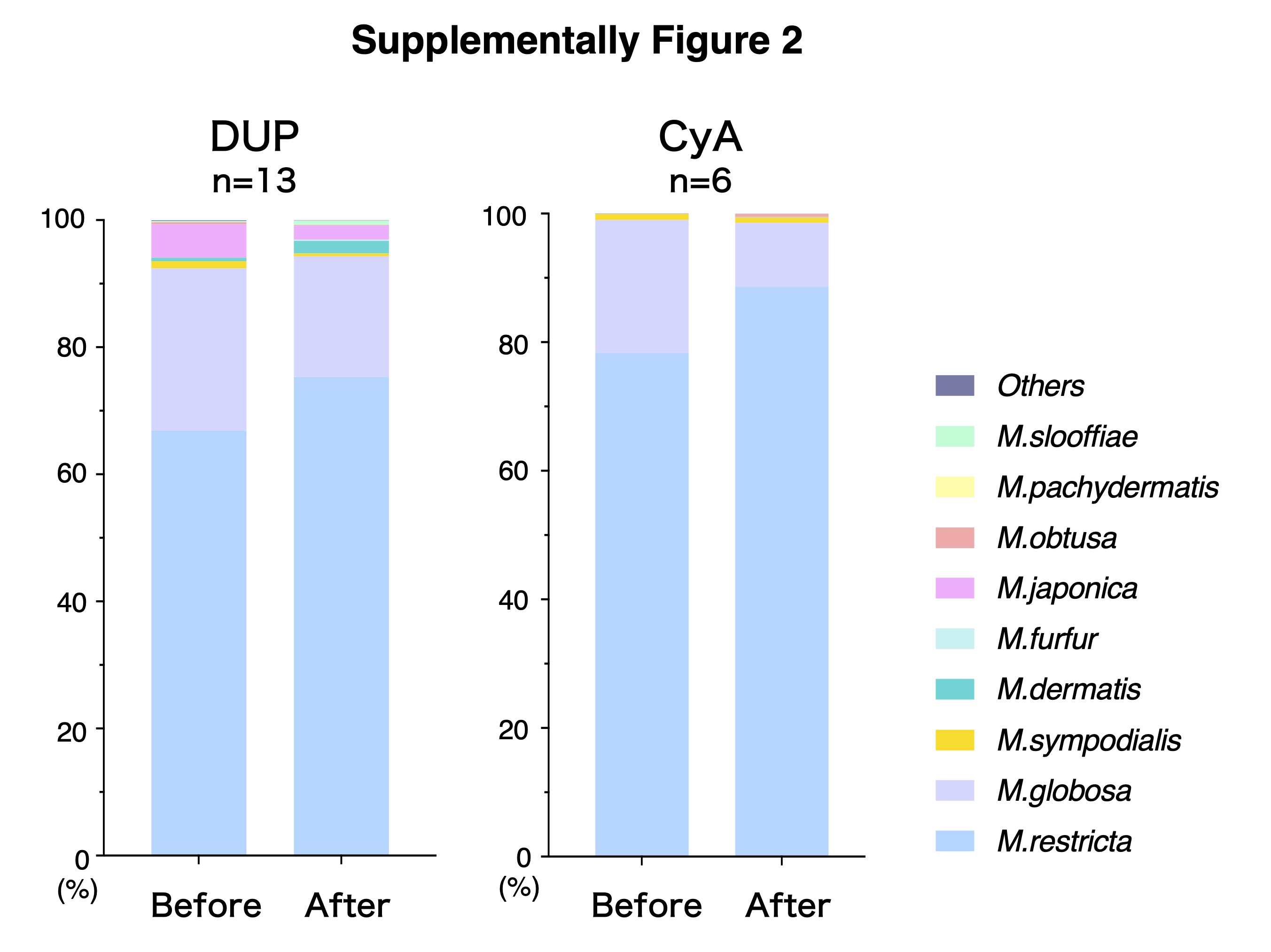

Supplement: Supplementary file 2 — Figure S2: Average proportion of fungal genera in DUP or CyA treatment groups. [file JDE-53-430-s004.jpg]

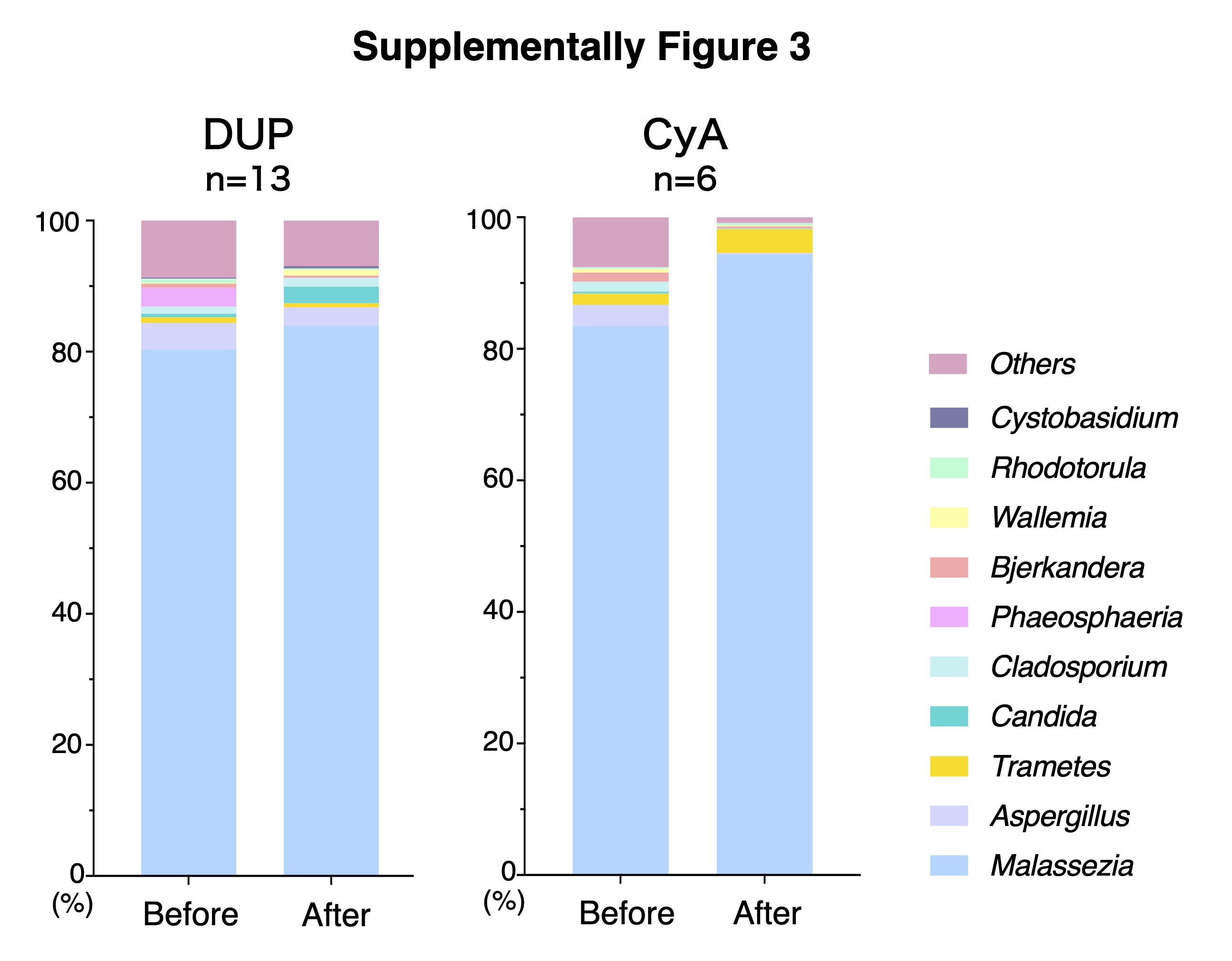

Supplement: Supplementary file 3 — Figure S3: Average proportion of Malassezia species in DUP or CyA treatment groups. [file JDE-53-430-s002.jpg]
