# Supplementary material for: Postauricular Skin Mycobiome Profiles in Atopic Dermatitis Treated With Dupilumab or Cyclosporine A: A Descriptive Case Series
Source: J Dermatol. 2025 Nov 28;53(3):430–6. doi: 10.1111/1346-8138.70083 (PMC12967678; doi:10.1111/1346-8138.70083)
Supplement: Supplementary file 4 — Table S1: List of detected fungal genera from skin swab samples. [file JDE-53-430-s003.docx]

**Supplementary Table 1**

| The name of detected genera | The average of the occupancy rate |
| --- | --- |
| Fungi;Basidiomycota;Malasseziomycetes;Malasseziales;Malasseziaceae;Malassezia | 0.843083459 |
| Fungi;Ascomycota;Eurotiomycetes;Eurotiales;Aspergillaceae;Aspergillus | 0.028763731 |
| Fungi;Basidiomycota;Agaricomycetes;Polyporales;Polyporaceae;Trametes | 0.013337575 |
| Fungi;Ascomycota;Saccharomycetes;Saccharomycetales;Debaryomycetaceae;Candida | 0.011097697 |
| Fungi;Ascomycota;Dothideomycetes;Capnodiales;Cladosporiaceae;Cladosporium | 0.010879042 |
| Fungi;Ascomycota;Dothideomycetes;Pleosporales;Phaeosphaeriaceae;Phaeosphaeria | 0.00975703 |
| Fungi;Basidiomycota;Agaricomycetes;Polyporales;Phanerochaetaceae;Bjerkandera | 0.006051021 |
| Fungi;Basidiomycota;Wallemiomycetes;Wallemiales;Wallemiaceae;Wallemia | 0.00444613 |
| Fungi;Basidiomycota;Microbotryomycetes;Sporidiobolales;Sporidiobolaceae;Rhodotorula | 0.003850729 |
| Fungi;Basidiomycota;Cystobasidiomycetes;Cystobasidiales;Cystobasidiaceae;Cystobasidium | 0.001950431 |
| Fungi;Basidiomycota;Agaricostilbomycetes;Agaricostilbales;Agaricostilbaceae;Sterigmatomyces | 0.001705612 |
| Fungi;Basidiomycota;Agaricomycetes;Polyporales;Meruliaceae;Phlebia | 0.001432512 |
| Fungi;Ascomycota;Eurotiomycetes;Eurotiales;Aspergillaceae;Penicillium | 0.001208735 |
| Fungi;Ascomycota;Saccharomycetes;Saccharomycetales;Trichomonascaceae;Trichomonascus | 0.000924715 |
| Fungi;Basidiomycota;Agaricomycetes;Russulales;Stereaceae;Stereum | 0.00090524 |
| Fungi;Basidiomycota;Tremellomycetes;Trichosporonales;Trichosporonaceae;Cutaneotrichosporon | 0.000889588 |
| Fungi;Basidiomycota;Tremellomycetes;Filobasidiales;Filobasidiaceae;Filobasidium | 0.000727187 |
| Fungi;Ascomycota;Dothideomycetes;Capnodiales;Mycosphaerellaceae;Mycosphaerella | 0.000701707 |
| Fungi;Ascomycota;Saccharomycetes;Saccharomycetales;Debaryomycetaceae;Meyerozyma | 0.000674207 |
| Fungi;Ascomycota;Dothideomycetes;Pleosporales;Phaeosphaeriaceae;Sclerostagonospora | 0.000642647 |
| Fungi;Basidiomycota;Tremellomycetes;Filobasidiales;Filobasidiaceae;Naganishia | 0.000620304 |
| Fungi;Ascomycota;Eurotiomycetes;Eurotiales;Trichocomaceae;Talaromyces | 0.000607699 |
| Fungi;Basidiomycota;Agaricomycetes;Corticiales;Corticiaceae;Hyphodontia | 0.000552557 |
| Fungi;Ascomycota;Dothideomycetes;Pleosporales;Pleosporaceae;Alternaria | 0.000526163 |
| Fungi;Basidiomycota;Agaricomycetes;Polyporales;Cerrenaceae;Cerrena | 0.00050983 |
| Fungi;Basidiomycota;Agaricomycetes;Agaricales;Physalacriaceae;Flammulina | 0.00044071 |
| Fungi;Ascomycota;Sordariomycetes;Myrmecridiales;Myrmecridiaceae;Myrmecridium | 0.000430208 |
| Fungi;Basidiomycota;Agaricomycetes;Polyporales;Fomitopsidaceae;Fomitopsis | 0.00042992 |
| Fungi;Ascomycota;Dothideomycetes;Capnodiales;Cladosporiaceae;Toxicocladosporium | 0.000399542 |
| Fungi;Ascomycota;Sordariomycetes;Hypocreales;Hypocreaceae;Trichoderma | 0.000377492 |
| Fungi;Ascomycota;Dothideomycetes;Pleosporales;Periconiaceae;Periconia | 0.00035734 |
| Fungi;Basidiomycota;Agaricomycetes;Polyporales;Phanerochaetaceae;Phlebiopsis | 0.00028326 |
| Fungi;Basidiomycota;Agaricomycetes;Agaricales;Omphalotaceae;Marasmiellus | 0.000281705 |
| Fungi;Basidiomycota;Agaricomycetes;Agaricales;Mycenaceae;Mycena | 0.000273972 |
| Fungi;Basidiomycota;Tremellomycetes;Tremellales;Bulleraceae;Bullera | 0.000272405 |
| Fungi;Ascomycota;Dothideomycetes;Capnodiales;Neodevriesiaceae;Neodevriesia | 0.000265234 |
| Fungi;Ascomycota;Sordariomycetes;Hypocreales;Nectriaceae;Corallomycetella | 0.000216491 |
| Fungi;Basidiomycota;Agaricomycetes;Russulales;Peniophoraceae;Peniophora | 0.000203591 |
| Fungi;Basidiomycota;Agaricomycetes;Agaricales;Schizophyllaceae;Schizophyllum | 0.000192895 |
| Fungi;Basidiomycota;Agaricomycetes;Russulales;Hericiaceae;Hericium | 0.000186515 |
| Fungi;Basidiomycota;Tremellomycetes;Cystofilobasidiales;Cystofilobasidiaceae;Cystofilobasidium | 0.000167489 |
| Fungi;Basidiomycota;Agaricomycetes;Polyporales;Irpicaceae;Emmia | 0.000138824 |
| Fungi;Basidiomycota;Agaricomycetes;Corticiales;Punctulariaceae;Punctularia | 0.000118887 |
| Fungi;Basidiomycota;Agaricomycetes;Atheliales;Atheliaceae;Athelia | 0.000115676 |
| Fungi;Basidiomycota;Agaricomycetes;Agaricales;Psathyrellaceae;Coprinellus | 0.000108114 |
| Fungi;Ascomycota;Eurotiomycetes;Eurotiales;Aspergillaceae;Phialosimplex | 9.89001E-05 |
| Fungi;Basidiomycota;Agaricomycetes;Polyporales;Irpicaceae;Irpex | 9.67181E-05 |
| Fungi;Basidiomycota;Agaricomycetes;Agaricales;Omphalotaceae;Gymnopus | 9.31674E-05 |
| Fungi;Basidiomycota;Exobasidiomycetes;Exobasidiales;Graphiolaceae;Graphiola | 8.34748E-05 |
| Fungi;Ascomycota;Dothideomycetes;Dothideales;Saccotheciaceae;Aureobasidium | 8.20214E-05 |
| Fungi;Ascomycota;Saccharomycetes;Saccharomycetales;Phaffomycetaceae;Cyberlindnera | 7.62603E-05 |
| Fungi;Ascomycota;Eurotiomycetes;Onygenales;Arthrodermataceae;Microsporum | 7.29596E-05 |
| Fungi;Basidiomycota;Agaricomycetes;Corticiales;Corticiaceae;Sistotrema | 6.97527E-05 |
| Fungi;Basidiomycota;Agaricomycetes;Agaricales;Lyophyllaceae;Hypsizygus | 6.91742E-05 |
| Fungi;Basidiomycota;Tremellomycetes;Tremellales;Bulleribasidiaceae;Vishniacozyma | 5.85844E-05 |
| Fungi;Basidiomycota;Agaricomycetes;Polyporales;Phanerochaetaceae;Phanerochaete | 5.42496E-05 |
| Fungi;Basidiomycota;Microbotryomycetes;Sporidiobolales;Sporidiobolaceae;Sporobolomyces | 5.17526E-05 |
| Fungi;Basidiomycota;Agaricomycetes;Agaricales;Physalacriaceae;Armillaria | 3.61447E-05 |
| Fungi;Ascomycota;Leotiomycetes;Helotiales;Rutstroemiaceae;Lanzia | 3.51427E-05 |
| Fungi;Ascomycota;Dothideomycetes;Pleosporales;Didymellaceae;Neoascochyta | 2.76743E-05 |
| Fungi;Ascomycota;Eurotiomycetes;Chaetothyriales;Cyphellophoraceae;Cyphellophora | 2.76743E-05 |
| Fungi;Ascomycota;Dothideomycetes;Pleosporales;Didymellaceae;Didymella | 2.64171E-05 |
| Fungi;Ascomycota;Sordariomycetes;Sordariales;Sordariaceae;Neurospora | 2.43941E-05 |
| Fungi;Ascomycota;Taphrinomycetes;Taphrinales;Taphrinaceae;Taphrina | 2.35868E-05 |
| Fungi;Basidiomycota;Agaricomycetes;Corticiales;Vuilleminiaceae;Australovuilleminia | 2.2993E-05 |
| Fungi;Ascomycota;Eurotiomycetes;Chaetothyriales;Herpotrichiellaceae;Exophiala | 2.26426E-05 |
| Fungi;Basidiomycota;Agaricomycetes;Agaricales;Psathyrellaceae;Coprinopsis | 2.25242E-05 |
| Fungi;Basidiomycota;Agaricomycetes;Polyporales;Polyporaceae;Coriolopsis | 1.86908E-05 |
| Fungi;Ascomycota;Dothideomycetes;Pleosporales;Phaeosphaeriaceae;Paraphoma | 1.70507E-05 |
| Fungi;Basidiomycota;Agaricomycetes;Corticiales;Corticiaceae;Peniophorella | 1.67787E-05 |
| Fungi;Basidiomycota;Ustilaginomycetes;Ustilaginales;Ustilaginaceae;Ustilago | 1.66268E-05 |
| Fungi;Basidiomycota;Agaricomycetes;Polyporales;Polyporaceae;Polyporus | 1.56742E-05 |
| Fungi;Ascomycota;Dothideomycetes;Pleosporales;Coniothyriaceae;Coniothyrium | 1.54099E-05 |
| Fungi;Basidiomycota;Agaricomycetes;Polyporales;Steccherinaceae;Steccherinum | 1.27275E-05 |
| Fungi;Ascomycota;Dothideomycetes;Capnodiales;Teratosphaeriaceae;Hortaea | 1.08499E-05 |
| Fungi;Basidiomycota;Agaricomycetes;Polyporales;Fomitopsidaceae;Piptoporus | 1.0236E-05 |
| Fungi;Ascomycota;Dothideomycetes;Capnodiales;Teratosphaeriaceae;Xenoteratosphaeria | 8.80547E-06 |
| Fungi;Ascomycota;Dothideomycetes;Pleosporales;Astrosphaeriellaceae;Pithomyces | 8.70005E-06 |
| Fungi;Ascomycota;Sordariomycetes;Hypocreales;Cordycipitaceae;Parengyodontium | 7.54755E-06 |
| Fungi;Ascomycota;Dothideomycetes;Pleosporales;Phaeosphaeriaceae;Parastagonospora | 6.65455E-06 |
| Fungi;Ascomycota;Dothideomycetes;Pleosporales;Pleosporaceae;Curvularia | 5.99254E-06 |
| Fungi;Ascomycota;Dothideomycetes;Pleosporales;Lophiostomataceae;Lophiostoma | 5.66732E-06 |
| Fungi;Basidiomycota;Agaricomycetes;Cantharellales;Botryobasidiaceae;Botryobasidium | 5.09881E-06 |
| Fungi;Ascomycota;Dothideomycetes;Pleosporales;Phaeosphaeriaceae;Phaeosphaeriopsis | 4.51742E-06 |
| Fungi;Ascomycota;Dothideomycetes;Pleosporales;Cucurbitariaceae;Neocucurbitaria | 4.40284E-06 |
| Fungi;Basidiomycota;Agaricomycetes;Polyporales;Panaceae;Panus | 3.30213E-06 |
| Fungi;Ascomycota;Saccharomycetes;Saccharomycetales;Metschnikowiaceae;Clavispora | 2.97387E-06 |
| Fungi;Ascomycota;Dothideomycetes;Capnodiales;Mycosphaerellaceae;Passalora | 1.96104E-06 |
| Fungi;Basidiomycota;Agaricomycetes;Russulales;Bondarzewiaceae;Heterobasidion | 1.96104E-06 |
